# Two decades taken at speed: genomics, cell biology, ecology, and evolution of protists

**DOI:** 10.1186/s12915-023-01787-9

**Published:** 2023-12-29

**Authors:** Joel B. Dacks, Michael L. Ginger

**Affiliations:** 1 Division of Infectious Diseases, Department of Medicine and Department of Biological Sciences, University of Alberta, 1-124 Clinical Sciences Building, 11350-83 Avenue, Edmonton, T6G 2G3 Canada; 2 Institute of Parasitology, Biology Centre, Czech Academy of Sciences, Branišovská 31, České Budějovice, 37005 Czech Republic; 3 Centre for Life’s Origins and Evolution, Department of Genetics, Evolution and the Environment, University College London, London, WC1E 6BT UK; 4 School of Applied Sciences, University of Huddersfield, Queensgate, Huddersfield, HD1 3DH UK

## Abstract

**In dynamic scientific fields, two decades can be an eternity, with technical and conceptual advances leading to drastically changed landscapes and paradigms. Noted natural philosopher Ferris Bueller once opined, “Life moves pretty fast. If you don’t look around once in a while, you could miss it”, and at the 20-year anniversary of** ***BMC Biology*, it is worth a “look around” at the field of evolutionary protistology. Things look quite differently today than they did when** ***BMC Biology* was founded.**

## The state of evolutionary protistology, then and now

Evolutionary protistology aims to determine the extent of eukaryotic phylogenetic diversity — the vast majority of which comprises protists, single-celled (or predominantly single-celled) eukaryotes — and to understand the evolutionary relationships among the major eukaryotic lineages. Having an accurate picture of this phylogenetic framework then impacts further aims of the field: framing evolution at deep and shallow time-points, contextualizing modern cell biology, understanding pathogenic mechanisms of infectious organisms, and assessing ecological and environmental change in oceans, rivers, lakes, and soils. Understanding has changed significantly in all these areas during the last two decades.

## 2003: the world that was

The early 2000s marked the end of a period of substantial upheaval within evolutionary protistology. A consensus had crystallized on the demise of the use of the term “Archezoa,” which had been a dominant paradigm suggesting that several eukaryotic lineages (including diplomonads, microsporidians, and parabasalids) had diverged from other eukaryotes prior to the acquisition of mitochondria. Instead, the concept of categorizing eukaryotic diversity into “super-groups” was on the horizon as it was recognized that all eukaryotes have — or had — a mitochondrion.

Coupled to advances in molecular phylogenetics, initially using hard won individual gene sequences (from EST libraries or degenerate PCR) and latterly from next-generation sequencing, this new concept of super groups sparked the realization that eukaryotic organelle diversity was far greater than previously imagined. Specifically, organelle degeneration and re-purposing contribute a larger role than previously anticipated in eukaryotic diversity, with organelles once inferred to have been absent in certain lineages, in fact being divergent or unrecognizable versions and hiding in plain view. This understanding was most prominently applied to mitochondria but turned out to be a characteristic of other organelles including Golgi bodies, flagella and centrioles, peroxisomes, and complex plastids, particularly as supergroups contain plastid and non-plastid possessing taxa alike. Moving through the mid-2000s, with no clear placement of the root of eukaryotes, evolutionary reconstructions moved to consensus building of the complement of proteins and features in the Last Eukaryotic Common Ancestor, e.g., [1]. Not that there were that many eukaryotic genomes to sift through — in 2003, the only eukaryotic microbes whose genomes had been sequenced were the yeast *Saccharomyces cerevisiae* and the malarial parasite *Plasmodium falciparum*, followed soon after by the diatom *Thalasiosira pseudonana*, the tropical disease parasites *Trypanosoma brucei*, *T. cruzi*, and *L. major* and the social amoeba *Dictyostelium discoideum*. It was just as well that in the broadest sense the genome sequences that became available early on covered nearly all of the then recognized supergroups. Thus began an explosive period of genomics, evolutionary protistology, and cell biology.

## Technology drives change

The last 20 years have been marked by an overwhelming abundance of genome and transcriptome data. During the early part of this era Sanger sequencing remained state of the art, but the advent of next-generation sequencing, and later long-read sequencing technology, opened avenues of inquiry previously unimaginable, whether chromosome-level assemblies of an individual genome or sampling of 100s of representatives to yield population-genomics scale datasets. The development of genetic tools for representatives across the tree of eukaryotes has allowed for deeply effective interplay between informatic and experimental biology, each providing iterative hypothesis generation and testing of the other. These tools enabled advances in, at least, three areas of particular relevance to the scope of BMC Biology.

## Marine protist ecology redefined

One of the most exciting developments in the last 20 years has been a deeper understanding of microbial eukaryotic ecology and the environment. Here, next-generation sequencing was applied to environmental DNA collected by ambitious multi-national cruises such as the Tara Oceans survey (2009–2013), which sampled the sunlit oceans at 100s of stations across the world, generating terabases of sequence data [2]. Including meta-barcoding, metagenomic, and meta-transcriptomic analysis to predict metabolism, these efforts mapped the biogeography of the major environmental players and provided insights into the physiological processes that drive the world’s oceans. As an example, diplonemids, then relatively unknown heterotrophic flagellates (mostly noted for their bizarre mitochondrial genome structures and previously considered to be trivial in an ecological context), were recognized from these global-scale surveys as highly abundant and possibly the most diverse group of marine planktonic eukaryotes [3]. These protists are present throughout the water column in all oceans, yet little remains known about their role(s) as key heterotrophic players in the largest ecosystem of the biosphere.

Improvements in the informatics of how microbial ecology is assessed, along with long-read technology for better sampling of meta-genomic and bar-coding, are now being leveraged by international collectives to produce fine-scale and accurate biogeographic maps of oceans, freshwater, and soils [4]. These will be invaluable in understanding, and perhaps even predicting or mitigating, the effects of climate change. However, refinements in traditional approaches to identifying microbial diversity cannot be dismissed, as recently illustrated by the identification and characterization of Provora, a new supergroup of predatory, eukaryovorous marine and freshwater flagellates. Numerically rare, genetically distinct from other eukaryotes, exhibiting novel feeding behavior and overlooked in molecular diversity surveys, provorans are nonetheless globally distributed. Their ecological role, perhaps as “top” microbial predators, remains to be explored [5].

## Molecular parasitology: the hunt for new interventions

For parasite studies, having a genome to hand provides insights into everything from genetic architecture and regulation to cell biology to evolution, but also identifies targets for chemotherapeutic intervention or allows understanding of mode of action for existing medicines. This is particularly true for medically important parasites that are refractory or challenging to culture to densities sufficient for biochemical studies. The malarial parasite *Plasmodium falciparum* provides an intriguing example.

Malaria and related apicomplexan parasites possess an essential, non-photosynthetic, and reduced chloroplast. This had been reported in the late 1990s, but the function of a plastid-derived organelle in a non-photosynthetic parasite was perplexing. In the early part of the last decade, elegant chemical rescue experiments revealed the sole essential function of this plastid in bloodstream malarial parasites to be biosynthesis of isoprenoid lipids through a pathway distinct from humans [6]. Plastid-null parasites generated by Yeh and DeRisi provide a powerful tool for continuing studies into drug and vaccine development. Looking forward, population genomics of parasites is a reality with its potential to reveal strain-level or biogeographical differences between parasites. There also remains a potential for parasite-specific proteins revealed from genome annotations to be applied in high-throughput screening for drug discovery. From an evolutionary perspective, parasitism is fundamentally a trophic strategy, one that evolved independently in different lineages from free-living ancestors. As more such free-living relatives of parasitic lineages are discovered and sequenced, it is becoming increasingly feasible to trace the changes in complements of molecular machinery underlying a given traits across the transition to parasitism in a given lineage. This can tell us the details of how the systems have changed in the individual instances of this switch in trophic strategy and perhaps even reveal common evolutionary drivers across eukaryotes.

## Evolutionary cell biology: an emerging field

Evolutionary protistology initially focused on endosymbiotic organelles such as mitochondria and plastids, largely because these were tractable with the microscopic and molecular biological tools then available. The past 20 years has seen the blossoming of evolutionary cell biology [7], driven by protist genome sequencing and a vastly more detailed understanding of the molecular machinery underpinning the cell biology of all manner of compartments and their variations. These include flagella, Golgi bodies, endosomes, peroxisomes, and the nucleus, together with how they interact.

Comparison across diverse eukaryotes can show if cellular systems identified in one supergroup (often animals or fungi) are restricted to it, or are present more broadly. Multi-disciplinary investigations concerning organellar function(s) and evolution are being reported (e.g., the recent study of mitochondria and peroxisome-related organelles in anaerobic amoebae [8]). Genome data are central to inferring cellular evolution but also unmask new molecular targets for study in additional organisms, be they animal, fungal, plant, or protist. Such examinations are becoming increasingly feasible due to the newly developed battery of genetic and cell biological tools in protist model organisms, many of which cell biologists working in yeast and mammalian models take for granted, and of the application to protists of systems-level tools such as Hyper-LOPIT proteomics. More tools for diverse protists are on the horizon.

## Into the next 20 years…

We have offered a sense of recent paradigm shifts in genome-led evolutionary protistology. But the community is clearly still in the discovery era, and it is difficult to predict what the landscape will look like 20 years from now. In the short-term, however, there are trends with momentum. Deeper sampling of protistan lineages across the tree of eukaryotes should allow for an ever more nuanced understanding of cellular history. Yet, genome sequences for organisms from some major lineages, such as ancyromonads, hemimastigiphorans, and provorans, remain to be obtained. These genomes will be critical to fully grasp the large-scale relationships among eukaryotes and how that relates to the deepest branches of the tree (i.e. the root), with all the inherent implications for understanding ancient eukaryotic history.

Techniques like single-cell RNA sequencing or systematic tagging of all proteins encoded in a genome [9] are just at the edge of broad-scale deployment. An age of truly comparative cell biology is in sight, bringing a more representative understanding of how eukaryotic cells work. Improved molecular evolutionary methods and sampling of diversity will certainly bring new ideas and models, leaving us closer to understanding our cellular past. New medicines or vaccines will likely continue to be required to treat long-established or newly emerging diseases caused by parasitic protists. Some protists offer generally unrealized potential for real-world applications in synthetic biology [10]. Moreover, Climate Crisis brings real urgency to investigation of protist ecology, diversity, and physiology. Sequencing capacity, computational power, and database representation have reached a stage where genome sequencing of mixed communities is no longer an unassailable obstacle. This is key as many unsequenced critical organisms must be cultured with prey, within microbial communities, or are in possession of symbionts. Community-level sequencing and ecology experiments such as RNA-stable isotope probing for microbial interactions will also be needed to understand the dynamics of our changing planet.

In the longer term, if the next 20 years are anything like the last, then we confidently expect that surprising and paradigm-shifting discoveries will be made. We cannot anticipate what many of these might be, but given the field’s powerful tools, and even more powerful curiosity, it should be an exciting period.

## Data Availability

Not applicable.
